# Trauma exposure, posttraumatic stress disorder and the effect of explanatory variables in paramedic trainees

**DOI:** 10.1186/1471-227X-14-11

**Published:** 2014-04-23

**Authors:** Celine B Fjeldheim, Jani Nöthling, Karin Pretorius, Marina Basson, Keith Ganasen, Robin Heneke, Karen J Cloete, Soraya Seedat

**Affiliations:** 1 Department of Psychiatry, University of Stellenbosch, Tygerberg, South Africa; 2 The Medical Research Council, University of South Africa Safety and Peace Promotion Research Unit, Parow, South Africa; 3 Department of Emergency Medical Sciences, Cape Peninsula University of Technology, Cape Town, South Africa

**Keywords:** Trauma, Posttraumatic stress disorder, Paramedic trainees, Emergency medical workers

## Abstract

**Background:**

Emergency healthcare workers, including trainees and individuals in related occupations are at heightened risk of developing posttraumatic stress disorder (PTSD) and depression owing to work-related stressors.

We aimed to investigate the type, frequency, and severity of direct trauma exposure, posttraumatic stress symptoms and other psychopathology amongst paramedic trainees. In order to create a risk profile for individuals who are at higher occupational risk of developing PTSD, we examined risk and resilience factors that possibly contributed to the presence and severity of posttraumatic symptomatology.

**Methods:**

Paramedic trainees (n = 131) were recruited from a local university. A logistic regression analysis was conducted using the explanatory variables age, gender, population group, trauma exposure, depression, alcohol abuse, alcohol dependence, resilience and social support.

**Results:**

94% of paramedic trainees had directly experienced trauma, with 16% meeting PTSD criteria. A high rate of depression (28%), alcohol abuse (23%) and chronic perceived stress (7%) and low levels of social support was found. The number of previous trauma exposures, depression, resilience and social support significantly predicted PTSD status and depression had a mediating effect.

**Conclusion:**

There is a need for efficient, ongoing screening of depressive and PTSD symptomatology in trauma exposed high risk groups so that early psychological supportive interventions can be offered.

## Background

Emergency Care Workers (ECW), for example police officers, fire fighters, rescue and disaster workers, military personnel and ambulance personnel, are at a higher occupational risk of developing posttraumatic stress disorder (PTSD) owing to their repeated exposure to critical incidents [1-5]. Critical incidents are events involving death, life-threatening injury or a crisis situation with a need for rescue or emergency that may result in stress-related reactions and the development of PTSD [6,7]. The mental health of ECW may be compromised by the nature of their work, which can be compounded by shorter recovery times [3]. ECW trainees may be at an even higher risk of developing PTSD due to exposure to a novel environment, age, inexperience in the field and the added pressure of academic evaluation [8]. However, few studies have investigated the prevalence and risk factors for PTSD in ECW trainees.

A South African study that investigated the relationship between exposure to critical incidents and prevalence of mental health problems among emergency medical care personnel (including traffic police, fire services, ambulance staff, and sea and air rescue workers) found that symptoms of anxiety, depression or PTSD intensified when exposure to critical incidents increased [9]. However, the rate at which symptoms increased eventually slowed over time, suggesting that there may be a time dependent desensitisation to the effects of repeated work-related traumatic exposures.

Comorbidity is another factor that may predispose ECW to subsequent PTSD. For example, in a UK study, researchers found that 10% of the 574 emergency medical care workers included in their study were suffering from clinical levels of depression and 22% met PTSD criteria [10]. Depression can also follow a traumatic event. For example, one study found that 16% of emergency personnel present at the scene of a tragic aeroplane crash were diagnosed with depression seven months after the incident [11]. Co-morbidity with depression may work bi-directionally, both as a possible pre-existing mediator for PTSD and also as a consequence or indirect effect of PTSD. In addition, high risk alcohol and drug use is positively correlated with PTSD symptomatology [12].

Several mitigating factors for PTSD in emergency workers and trainees have been identified. For example, social support following a traumatic incident has been found to be protective against PTSD [8,13,14]. In one study, ECW who reported strong peer support also reported lower levels of perceived stress [15]. Similarly, a Dutch study found that lack of social support from supervisors greatly compounded symptoms of burnout in ambulance personnel [16]. Resilience, the ability to cope with adversity without being harmed by it, can also buffer against the onset of PTSD symptoms, and resilience-recovery variables (personality, coping strategies and social support) can aid in trauma adjustment [17,18].

ECW are exposed to living individuals who are seriously injured or dying and, as such, are exposed to human pain and suffering [2]. They have to make quick appraisals and administer aid in an attempt to save lives, often without support or reassurance [4]. ECW workers are frequently unaware of the specifics of the emergency situation as they are often the first personnel on site and consequently do not have adequate time or information to mentally prepare themselves. Due to the nature of their work, ECW face an increased risk of developing PTSD symptoms.

The purpose of this study was firstly to assess and determine the frequency, nature and severity of direct trauma exposure, posttraumatic stress symptoms and other psychopathology amongst paramedic trainees. Secondly, the study aimed to identify risk factors (e.g. trauma exposure) and resilience factors (e.g. social support) that contribute to the presence and severity of posttraumatic symptomatology amongst paramedic trainees. Thirdly, the study aimed to create a risk profile for paramedic trainees who are at higher occupational risk of developing PTSD.

## Methods

### Participants and procedure

A hundred and thirty one paramedic trainees participated in the study. Data were collected between 2008 and 2011. Participants were recruited from a university in the Western Cape with the aid and permission of their supervisors. All the participants were first year paramedic trainees. During their first year of study, trainees gain practical experience in the field and are exposed to accident scenes and critical incidents.

The study was approved by the Health Research Ethics Committee at Stellenbosch University, Cape Town, South Africa (N06/02/037). Informed consent was obtained from participants before assessment commenced. Two researchers (a psychiatry resident and research psychologist) conducted the assessments. Participants were assessed with a battery of questionnaires including a demographic questionnaire as well as several measures of psychiatric and psychological status.

### Measures

#### Life Events Checklist (LEC)

Trauma exposure was measured using the Life Events Checklist (LEC), a questionnaire from the Clinician-Administered PTSD Scale for DSM-IV (CAPS) [19]. This scale assesses the direct and vicarious experience of various traumatic events, and it has demonstrated adequate psychometric properties [19]. A total score can be computed to reflect the number of different types of traumatic events experienced; the LEC does reflect the number of exposures per event. If two or more significant traumatic incidences are recorded, the participant is required to identify the one that is most severe.

#### Davidson Trauma Scale (DTS)

The Davidson Trauma Scale (DTS) assesses the frequency and severity of PTSD symptoms according to PTSD criteria in the DSM-IV-TR, and has been used as a symptom status measure in various populations [20]. Avoidance and intrusion elements are explored with reference to the traumatic event, whereas components relating to numbing, hyper-arousal and withdrawal are assessed as present or absent without direct affixation to the event in question [21]. The questionnaire consists of 17 items, measured on a scale of 0 – 4 for frequency as well as severity. A cut-off score of 40 is considered to indicate the presence of PTSD. The DTS has shown good test-retest reliability (*r* = 0.86) and internal consistency (*r* = 0.99), as well as good validity.

#### Centre for Epidemiological Studies Depression Scale (CES-D)

Participants were screened for depression with the Centre for Epidemiologic Studies Depression Scale (CES-D). The CES-D has a 5-point Likert scale. Scores on all items are added together. A score of 16 or more indicates possible depression i.e. a score of 16 or more is the cut-off for depression. The scale has very good reliability, with a Cronbach’s alpha value of 0.85 in the general population and 0.90 in a psychiatric population [22]. In a South African study, to examine cross-cultural relevance, a Cronbach’s α of 0.90 was found [23].

#### Alcohol Use Disorders Identification Test (AUDIT)

The Alcohol Use Disorders Identification Test (AUDIT) was used to identify participants with problematic alcohol use. It consists of 10 items relating to alcohol usage and responses are rated on a 5-point Likert scale. A score of 8 or higher indicates possible alcohol abuse. This scale has shown good reliability and validity, and has been tested in various cultures [24].

#### Patient Health Questionnaire (PHQ-15)

The Patient Health Questionnaire (PHQ-15) was used to assess participants’ perceptions of their physical health. Responses are measured on a 3-point Likert scale. This 15-item questionnaire has a cut-off score of 15 or more for high symptom severity, and has displayed good internal reliability with a Cronbach’s alpha value of 0.80 [25].

#### Perceived Stress Scale (PSS-10)

The 10-item Perceived Stress Scale (PSS-10) was used to assess participants’ perceptions of stress. It is a 10-item questionnaire measured on a 4-point Likert scale and has good reliability and validity [26]. The PSS-10 measures the degree to which situations in one’s life are appraised as stressful. Items are designed to tap how unpredictable, uncontrollable, and overloaded respondents find their lives. The scale also includes a number of direct queries about current levels of experienced stress.

#### Multidimensional Scale of Perceived Social Support (MSPSS)

The degree of social support was measured with the Multidimensional Scale of Perceived Social Support (MSPSS) which measures participants’ own perception of social support. The questionnaire consists of 12 items relating to support from family, friends, or a significant other, and is rated on a 7-point Likert scale. It has good reliability, factorial validity as well as construct validity [27].

#### Connor-Davidson Resilience Scale (CD-RISC)

The Connor-Davidson Resilience Scale (CD-RISC) is a 25-item questionnaire with each item scored on a 5-point Likert scale. The scale measures resilience based on five factors: (1) personal competence, high standards and tenacity; (2) trust in one’s own instincts, tolerance of negative affect and strengthening effects of stress; (3) social support; (4) control and; (5) spiritual influences e.g. faith [28]. The scale has a range of 0–100, with higher scores reflecting greater resilience. It has good reliability (Cronbach’s α = 0.89) and strong validity [28].

### Data analysis

The data were analysed using SPSS, version 20. Frequencies and score means were obtained for demographic variables, and reliability tests were conducted for each scale to obtain a Cronbach’s alpha value [29]. The normality distribution of the data was examined with Kolmogorov-Smirnov tests. Mann–Whitney U tests were conducted to assess for differences based on PTSD status, on variables of direct trauma exposure, depression, alcohol abuse, physical health symptoms, perceived stress, social support and resilience. Logistic regression models were used to determine the predictive value of demographic characteristics and mental health measures on PTSD status. Gender, age, population group and level of exposure were entered into the first model, followed by the addition of depression, perceived stress and alcohol use or abuse in model 2 and the addition of perceived social support and resilience in model 3.

## Results

### Sample demographics

Participants were mainly of White (47%) and Coloured (33%; the term Coloured is a demographic marker that historically denotes South Africans of African, European and/or Asian ancestry) population groups and were predominantly male (64%). The mean age of participants were 22 (range: 17–39). The majority of participants were not married (92%). The demographic characteristics and prevalence of psychiatric disorders are presented in Table 1.

**Table 1:** Demographic characteristics of the sample

	**N**	**%**	**M**
Gender	132		
Male	84	63.6	
Female	48	36.4	
Age	129*		22.05
Population group	132		
Black	18	13.6	
Coloured	44	33.3	
White	62	47.0	
Indian	5	3.8	
Other	1	0.8	

*missing data.

### Data screening

Kolmogorov-Smirnov tests were used to determine the normality distribution of the data. The Kolmogorov-Smirnov tests were significant for the variables PTSD, depression, alcohol abuse, physical health status, perceived stress and level of exposure indicating that the data for these variables were not normally distributed. Resilience was the only variable with a non-significant Kolmogorov-Smirnov statistic and therefore the only normally distributed variable. Mann–Whitney tests and logistic regression, methods not dependent on normal distribution, were therefore used to analyse the data.

Cronbach’s alpha for the DTS indicated excellent reliability (α = 0.96). Other scales also yielded excellent reliability: the CD-RISC (α = 0.92), the MSPSS (α = 0.93), CES-D (α = 0.91) and AUDIT (α = 0.87). The PSS-10 and the PHQ had alpha values of 0.75 and 0.78, respectively, which are regarded as acceptable [29].

### Trauma exposure and PTSD status

The majority of participants (94%) had directly experienced a lifetime traumatic event. Traumatic events that were directly experienced were incidents that had either happened to the participant or had been witnessed. The most commonly endorsed traumas were witnessing a transport accident (53%), sudden unexpected death of someone close (51%), witnessing someone suffering from a life-threatening illness/injury (51%), witnessing a fire/explosion (39%), and being the victim of a physical assault (33%). Among those with PTSD, the most commonly endorsed trauma was witnessing a transport accident (n = 15, 65%).

### Mental health, physical health and PTSD status

High prevalence rates were found for PTSD (16%), depression (28%), alcohol abuse (24%) and alcohol dependence (8%). Participants meeting PTSD criteria had significantly higher levels of trauma exposure *U*(124) = 634.5, *p* = .003, depression *U*(122) = 188, *p* < .000, perceived stress *U*(122) = 439, *p* < .000 and physical health symptoms *U*(123) = 437, *p* < .000 compared to participants who did not meet criteria for PTSD. Participants meeting PTSD criteria also had significantly lower levels of resilience *U*(122) = 656.5, *p = .*012 and social support *U*(124) = 682, *p* = .008. There was no significant difference in alcohol abuse *U*(124) = .154, *p* = .154 scores between participants meeting PTSD criteria and participants not meeting the criteria. Results are presented in Table 2.

**Table 2:** Comparison of mental and physical health measures of participants based on PTSD status

	**With PTSD**			**Without PTSD**			**U**	**df**	**p**
	**n**	**Mean rank**	**Sum of ranks**	**n**	**Mean rank**	**Sum of ranks**			
Trauma exposure	21	83.79	58.16	103	58.16	5999.50	634.5	124	.003
Depression (CES-D)	21	103.05	2164	101	52.86	5339	188	122	.000
Perceived stress (PSS-10)	21	91.07	1912.5	101	55.35	5590.5	439	122	.000
Physical health symptoms (PHQ-15)	21	92.19	1936	102	55.78	5690	437	123	.000
Resilience (CDRISC)	20	43.33	866.50	102	65.06	6636.50	656.5	122	.012
Social support (MSPSS)	21	43.48	913	103	66.38	6837	682	124	.008
Alcohol abuse (AUDIT)	21	72.52	1523	103	60.46	6227	871	124	.154

### Predictors of PTSD status

Multivariate logistic regression was used to assess explanatory variables of PTSD. The regression model included demographic variables and mental health variables, with PTSD status as the dependent variable. The following variables were entered into the first regression model: age, gender, population group and number of total previous trauma exposures. In model 2 alcohol abuse, alcohol dependence, perceived stress and depression where added to the demographic variables and trauma exposure in model 1. Model 3 contained the variables used in model 1 and model 2 with the addition of social support and resilience. In model 1, with only the demographic variables and trauma exposure included to assess their effects on PTSD status, only number of previous trauma exposures significantly predicted PTSD status (*β* = .24, *p* = .003, OR 1.27). In model 2, with the demographic variables, trauma exposure, perceived stress, depression and alcohol abuse/dependence, only depression significantly predicted PTSD status (*β* = .21, *p* = .003, OR 1.23) and number of previous trauma exposures was no longer a significant predictor (*β* = .15, *p* = .184, OR 1.16). In model 3, with the demographic variables, trauma exposure, perceived stress, depression, alcohol abuse/dependence and social support and resilience, depression (*β* = .335, *p* = .002, OR 1.40), social support (*β* = −.74, *p* = .020, OR .93) and resilience (*β* = .114, *p* = .020, OR 1.12) significantly predicted PTSD status. Results are presented in Table 3.

**Table 3:** Parameters for the variables predicting PTSD status

							**95% confidence interval**	
**Model**		**B**	**Std. error**	**Wald’s χ**^**2**^	**Sig.**	**Exp(B) odds ratio**	**Lower limit**	**Upper limit**
1	Age	-.12	.09	1.88	.171	.89	.75	1.05
	Gender	-.20	.58	.12	.731	.82	.24	2.57
	Population group				.754			
	Trauma exposure (LEC)	.24	.08	8.90	.003*	1.27	1.09	1.49
2	Age	-.32	.18	3.41	.065	.72	.51	1.02
	Gender	.09	.87	.01	.919	1.09	.120	6.05
	Population group				.940			
	Trauma exposure (LEC)	.15	.11	1.77	.184	1.16	.93	1.43
	Perceived stress (PSS-10)	-.02	.09	.04	.834	.98	.83	1.16
	Alcohol abuse (AUDIT)	-.90	1.08	.70	.404	.41	.05	3.39
	Alcohol dependence (AUDIT)	.29	1.57	.04	.852	1.34	.06	28.84
	Depression (CESD)	.21	.07	8.75	.003*	1.23	1.07	1.42
3	Age	-.36	.19	3.66	.056	.70	.48	1.01
	Gender	.37	1.04	.13	.723	1.45	.19	11.15
	Population group			1.34	.855			
	Trauma exposure (LEC)	.14	.12	1.34	.248	1.15	.91	1.46
	Perceived stress (PSS-10)	.04	.10	.19	.667	1.05	.86	1.28
	Alcohol abuse (AUDIT)	−2.42	1.28	3.56	.059	.09	.01	1.1
	Alcohol dependence (AUDIT)	.76	1.75	.19	.666	2.13	.07	65.43
	Depression (CESD)	.34	.11	9.83	.002*	1.40	1.13	1.73
	Social support (MSPSS)	-.07	.03	5.43	.020*	.93	.87	.99
	Resilience (CDRISC)	.11	.05	5.41	.020*	1.12	1.02	1.24

*p < .05.

## Discussion

The main findings of this study can be summarised as follows: paramedic trainees had high rates of PTSD, depression and trauma exposure (based on self-reported symptoms). Participants meeting criteria for PTSD had significantly higher rates of depression, perceived stress and physical health symptoms and significantly lower rates of resilience and social support. Higher rates of trauma exposure and depression and lower rates of social support and resilience were significant predictors of PTSD. Depression had a mediating effect on the relationship between trauma exposure and PTSD. These finding mirrors prior research [10,11].

Paramedic trainees had high rates of trauma exposure, both related (e.g. witnessing a transport accident) and unrelated (e.g. being a victim of physical assault) to work. Trauma exposure that is unrelated to work, including childhood exposure to violence, abuse and neglect, may influence career choice among paramedics. A high rate (38.4%) of physical, sexual and emotional abuse was found in a sample of Canadian veteran paramedics [30]. An association between childhood abuse and neglect and higher mental and physical health symptom scores was also reported in that sample [30]. Together with the findings of our study, it suggests that exposure to early adversity may impact on the career choice of paramedics.

16% of paramedic trainees met symptom criteria for PTSD. The rate of current PTSD is considerably higher than the 12 month prevalence rate of 0.6% among South Africans (based on lay administered structural interview) [31]. The rate of PTSD is consistent with that documented by a group of Dutch researchers (2003) who found that 12% of emergency workers displayed PTSD symptoms [16]. Two other studies found much higher rates of PTSD symptomatology among ambulance service workers at 21% and 22%, respectively [10,32].

The current study also found high rates of depression among paramedic trainees (28%). Depression was a significant predictor of PTSD and had a mediating effect between trauma exposure and PTSD status. In a study of disaster workers 16% of workers developed depression seven months after work-related trauma exposure [11]. The rate of depression among emergency ambulance workers in the UK has been found to approximate 10% [10]. Differences in the rate of depression may, in part, be due to ascertainment differences or secondary to the high rates of exposure to trauma in South Africa.

Rates of alcohol abuse were similarly high with 24% of paramedic trainees meeting criteria for abuse. Twelve month prevalence rates of alcohol abuse in the South African general population have been estimated at 4.5% and 11.1 for life-time prevalence in the age group 18–34 (based on a lay administered structured interview) [30]. A study conducted in the South African higher education sector found that 11% of students in the age group 15–49 consumed alcohol on a weekly or daily basis [33]. This suggests a disturbing pattern of alcohol abuse within at-risk vocations. Males had higher rates of alcohol abuse than females. These findings are in line with the South African Stress and Health study where substance use disorders were found to be significantly associated with male gender. Alcohol abuse in emergency medical care occupations should be investigated further, given the high prevalence of alcohol abuse in the Western Cape Province of South Africa [34].

In a Brazilian study it was found that ambulance workers with PTSD had significantly poorer physical and mental health than workers without PTSD [35]. This, too, was the case in the current study, with the PTSD group endorsing more physical health ailments than the non-PTSD group. Paramedic trainees with PTSD had a higher mean number of varied traumatic exposures, higher levels of depression and stress, poorer physical health and lower levels of social support and resilience than those without PTSD. Previous studies have also shown that higher trauma exposure, stress and depression levels, low resilience and low social support are associated with PTSD [18,36,37].

Overall, resilience and social support were predictors of PTSD status. We measured social support with regards to three areas of personal contact – family, friends and a significant other. Social support may be conceptualised on three tiers, namely support by family and friends (tier one), support by community and religious organisations (tier two) and support by formal services such as the police (tier three) [38]. Social support is considered to improve coping, decrease stress levels and has a positive effect on health and well-being [38]. In a Dutch study, social support in the workplace was found to positively predict PTSD in emergency care personnel [16]. It has been proposed that the most common type of social support associated with PTSD is emotional support: the more emotional support received (from loved ones or supervisors and colleagues) after a traumatic event, the lower the risk of developing PTSD [38]. Many studies have focused on support provided in the workplace or by emergency service staff rather than on personal social support, which could explain some of the discrepancy across studies on the effects of social support on PTSD [14,16,39].

### Strengths and limitations

Some limitations deserve mention. Since the measures employed were self-report questionnaires, the responses reflect the participants’ perceptions and not clinician or trained lay interviewer diagnoses. The use of self-report measures may have inflated the frequency of psychiatric disorders found in this sample. Participants reported experiencing traumas unrelated to their occupation which may have contributed to PTSD symptomatology. The large number of questionnaires administered in one sitting could have caused participant fatigue and this may have influenced the accuracy of the results. The study was also cross-sectional in design which precludes causal inferences and measurement of symptom change over time. The cross-sectional design also limits the interpretation of the mediation analysis. We cannot determine if the mediating effect is due to comorbidity (e.g. depression and PTSD) or if there is a temporal sequence of events (e.g. trauma leads to depression and depression leads to PTSD).

Several aspects of the sample distinguish this study from previous research. While studies have investigated PTSD among paramedic staff in South Africa, none, to our knowledge, have investigated predictors of PTSD among paramedic trainees. Trauma exposure is common among paramedic staff and trainees are particularly vulnerable to the adverse effects associated with trauma exposure, due to a lack of experience. Early identification and treatment of PTSD is important to prevent chronic PTSD and the debilitating effects thereof. The homogeneity of the sample is an added strength as there have been few studies on risk factors for PTSD that focus on specific trauma types and at-risk populations. Future studies could compare the effects of trauma frequency and repeated same-trauma exposures on mental and physical health outcomes in paramedic trainees and practising, experienced paramedics, as well as include other occupation groups, such as police officers and fire fighters.

## Conclusion

In conclusion, there is a need to better understand risk and mitigating factors for PTSD in high-risk occupational groups. The results of this study indicate that paramedic trainees have high rates of PTSD and those who meet PTSD criteria have higher rates of perceived stress and depression, lower rates of social support and resilience, and poorer physical health, which can be detrimental to overall health. The study findings also suggest that depression is a mediating factor for PTSD and social support and resilience are significant predictors of PTSD.

The need for efficient screening of PTSD and depression symptomatology in trauma-exposed high risk groups needs to be emphasized so that targeted psychological and supportive interventions, initiated early and continued over time, can be offered. Paramedic trainees who have been exposed to multiple traumas and who have poor social support and resilience may be in need of preventative psychotherapeutic interventions in the workplace to offset the development of PTSD and other psychiatric sequelae. While paramedic trainees may need additional support, it should be noted that they were exposed to work-related traumatic events over a relatively short time period. With the passage of time and accumulation of experience, these trainees may show adequate psychological adjustment in this setting. These results are arguably generalisable to paramedic trainees beyond South Africa as these occupational groups bear many similarities with regards to work-related trauma, regardless of setting.

## Competing interests

The authors declare that they have no competing interests.

## Authors’ contributions

CBF participated in data collection, analysis of data, interpretation of results and drafting of the manuscript. JN participated in the analysis of the data, interpretation of results and drafting of the manuscript. KP participated in the analysis of the data, interpretation of results and drafting of the manuscript. MB participated in data collection, analysis of data and interpretation of results. KG participated in the study conception and design, data collection and critical revision of the drafted manuscript RB participated in the study design and data collection. KJC participated in data analysis and interpretation of results and critical revision of the drafted manuscript. SS participated in the conception and design of the study, analysis of data, interpretation of results and critical revision of the drafted manuscript. All authors read and approved the final manuscript.

## Pre-publication history

The pre-publication history for this paper can be accessed here:

http://www.biomedcentral.com/1471-227X/14/11/prepub

## References

[B1] Jonsson A Segesten K Mattsson B Post-traumatic stress among Swedish ambulance personnel Emerg Med J 2003 20 79 84 10.1136/emj.20.1.79 12533382 PMC1726002

[B2] Regehr C Goldberg G Hughes J Exposure to human tragedy, empathy, and trauma in ambulance paramedics Am J Orthopsychiatry 2002 72 4 505 513 15792036 10.1037/0002-9432.72.4.505

[B3] Alexander DA Klein S Ambulance personnel and critical incidents: impact of accident and emergency work on mental health and emotional well-being Br J Psychiatry 2001 178 76 81 10.1192/bjp.178.1.76 11136215

[B4] Beaton R Murphy S Johnson C Pike K Corneil W Exposure to duty-related incident stressors in urban fire fighters and paramedics J Trauma Stress 1998 11 4 821 828 10.1023/A:1024461920456 9870232

[B5] Schiraldi GR The Post-Traumatic Stress Disorder Sourcebook (Second Edition) 2009 USA: McGraw Hill

[B6] Lane P Critical incident stress debriefing for health care workers Omega: J Death Dying 1993 28 301 315

[B7] Walker G Crisis-care in critical incident debriefing Death Stud 1990 14 2 121 133 10.1080/07481189008252354

[B8] Lowery K Stokes MA Role of peer support and emotional expression on posttraumatic stress disorder in student paramedics J Trauma Stress 2005 18 2 171 179 10.1002/jts.20016 16281211

[B9] Ward CL Lombard CJ Gwebushe N Critical incident exposure in South African emergency services personnel: prevalence and associated mental health issues Emerg Med J 2006 23 226 231 10.1136/emj.2005.025908 16498167 PMC2464423

[B10] Bennett P Williams Y Page N Hood K Woollard M Levels of mental health problems among UK emergency ambulance workers Emerg Med J 2004 21 235 236 10.1136/emj.2003.005645 14988360 PMC1726283

[B11] Fullerton CS Ursano RJ Wang L Acute stress disorder, posttraumatic stress disorder, and depression in disaster or rescue workers Am J Psychiatry 2004 161 8 1370 1376 10.1176/appi.ajp.161.8.1370 15285961

[B12] Donnelly E Siebert D Occupational risk factors in the emergency medical services Prehosp Disaster Med 2009 24 422 429 20066645 10.1017/s1049023x00007251

[B13] Yehuda R Risk and resilience in posttraumatic stress disorder J Clin Psychiatry 2004 65 Suppl 1 29 36 14728094

[B14] Brewin CR Andrews B Valentine JD Meta-analysis of risk factors for posttraumatic stress disorder in trauma-exposed adults J Consult Clin Psychol 2000 68 5 748 766 11068961 10.1037//0022-006x.68.5.748

[B15] Revicki DA Whitley TW Gallery ME Organizational characteristics, perceived work stress, and depression in emergency medicine residents Behav Med 1993 19 2 74 81 10.1080/08964289.1993.9937568 8280965

[B16] Van der Ploeg E Kleber RJ Acute and chronic job stressors among ambulance personnel: predictors of health symptoms Occup Environ Med 2003 60 40 46 10.1136/oem.60.suppl_1.i40 PMC1765729 12782746

[B17] Davydov DM Steward R Ritchie K Chaudieu I Resilience and mental health Clin Psychol Rev 2010 30 479 495 10.1016/j.cpr.2010.03.003 20395025

[B18] Lawrence JW Fauerbach JA Personality, coping, chronic stress, social support and PTSD symptoms among adult burn survivors: a path analysis J Burn Care Rehabil 2003 24 1 63 72 10.1097/00004630-200301000-00016 12543997

[B19] Gray MJ Litz BT Hsu JL Lombardo TW Psychometric properties of the life events checklist Assessment 2004 11 4 330 341 10.1177/1073191104269954 15486169

[B20] Davidson JRT Tharwani HM Connor KM Davidson Trauma Scale (DTS): normative scores in the general population and effect sizes in placebo-controlled SSRI trials Depress Anxiety 2002 15 75 78 10.1002/da.10021 11891997

[B21] Davidson JRT Book SW Colket JT Tupler LA Roth S David D Hertzberg M Mellman T Beckham JC Smith RD Davison RM Assessment of a new self-rating scale for post-traumatic stress disorder Psychol Med 1997 27 153 160 10.1017/S0033291796004229 9122295

[B22] Radloff LS The CES-D scale: a self-report depression scale for research in the general population Appl Psychol Meas 1977 1 3 385 401 10.1177/014662167700100306

[B23] Pretorius B Cross-cultural application of the center for epidemiological studies depression scale: a study of Black South African students Psychol Rep 1991 69 1179 1185 10.2466/pr0.1991.69.3f.1179 1792288

[B24] Babor TF Higgins-Biddle JC Saunders JB Monteiro MG AUDIT: The Alcohol Use Disorders Identification Test: Guidelines for use in primary care (Second Edition) 2001 Geneva: World Health Organization

[B25] Kroenke K Spitzer RL Williams JBW The PHQ-15: validity of a new measure for evaluating the severity of somatic symptoms Psychosom Med 2002 64 258 266 11914441 10.1097/00006842-200203000-00008

[B26] Cohen S Williamson G Spacapan S, Oskamp S Perceived stress in a probability sample of the United States. The social psychology of health 1988 Newbury Park, CA: Sage 31 67

[B27] Zimet GD Dahlem NW Zimet SG Farley GK The multidimensional scale of perceived social support J Pers Assess 1988 52 1 30 41 10.1207/s15327752jpa5201_2 2280326

[B28] Connor KM Davidson JRT Development of a new resilience scale: the Connor-Davidson Resilience Scale (CD-RISC) Depress Anxiety 2003 18 76 82 10.1002/da.10113 12964174

[B29] Bland JM Altman DG Statistics notes: Cronbach’s alpha BMJ 1997 314 572 10.1136/bmj.314.7080.572 9055718 PMC2126061

[B30] Maunder RG Halpern J Schwartz B Gurevich M Symptoms and responses to critical incidents in paramedics who have experienced childhood abuse and neglect Emerg Med J 2012 29 222 227 10.1136/emj.2010.099838 21422029

[B31] Herman AA Stein DJ Seedat S Heeringa SG Moomal H Williams DR The South African Stress and Health (SASH) study: 12-month and lifetime prevalence of common mental disorders S Afr Med J 2009 99 5 339 344 19588796 PMC3191537

[B32] Clohessy S Ehlers A PTSD Symptoms, response to intrusive memories and coping in ambulance service workers Br J Clin Psychol 1999 38 251 265 10.1348/014466599162836 10532147

[B33] HEAIDS HIV prevalence and related factors – higher education sector, South Africa, 2008–2009 2010 Pretoria: Higher education South Africa

[B34] Stein DJ Seedat S Herman A Moomal H Heeringa SG Kessler RC Williams DR Lifetime prevalence of psychiatric disorders in South Africa Br J Psychiatry 2008 192 112 117 10.1192/bjp.bp.106.029280 18245026 PMC2718689

[B35] Berger W Figueira I Maurat AM Bucassio EP Vieira I Jardim SR Coutinha ES Mari JJ Mendlowicz MV Partial and full PTSD in Brazilian ambulance workers: prevalence and impact on health and on quality of life J Trauma Stress 2007 20 4 637 642 10.1002/jts.20242 17721969

[B36] Connor KM Davidson JRT Lee LC Spirituality, resilience, and anger in survivors of violent trauma: a community survey J Trauma Stress 2003 16 5 487 494 10.1023/A:1025762512279 14584633

[B37] Holeva V Tarrier N Wells A Prevalence and predictors of acute stress disorder and PTSD following road traffic accidents: thought control strategies and social support Behav Ther 2001 32 1 65 83 10.1016/S0005-7894(01)80044-7

[B38] Letvak S The importance of social support for rural mental health Issues Ment Health Nurs 2002 23 249 261 10.1080/016128402753542992 11942190

[B39] Ozer EJ Best SR Lipsey TL Weiss DS Predictors of posttraumatic stress disorder and symptoms in adults: a meta-analysis Psychol Bull 2003 129 1 52 73 12555794 10.1037/0033-2909.129.1.52

